# Supplementary material for: Pollinator and host sharing lead to hybridization and introgression in Panamanian free‐standing figs, but not in their pollinator wasps
Source: Ecol Evol. 2023 Jan 18;13(1):e9673. doi: 10.1002/ece3.9673 (PMC9848820; doi:10.1002/ece3.9673)
Supplement: Supplementary file 1 — supplementary [file ECE3-13-e9673-s001.pdf]

Supplemental Information for:

Pollinator and host sharing lead to hybridization and introgression in Panamanian free-standing figs, but not in their pollinator wasps

Jordan D. Satler, Edward Allen Herre, Tracy A. Heath, Carlos A. Machado, Adalberto Gómez Zúñiga, K. Charlotte Jandér, Deren A. R. Eaton, and John D. Nason

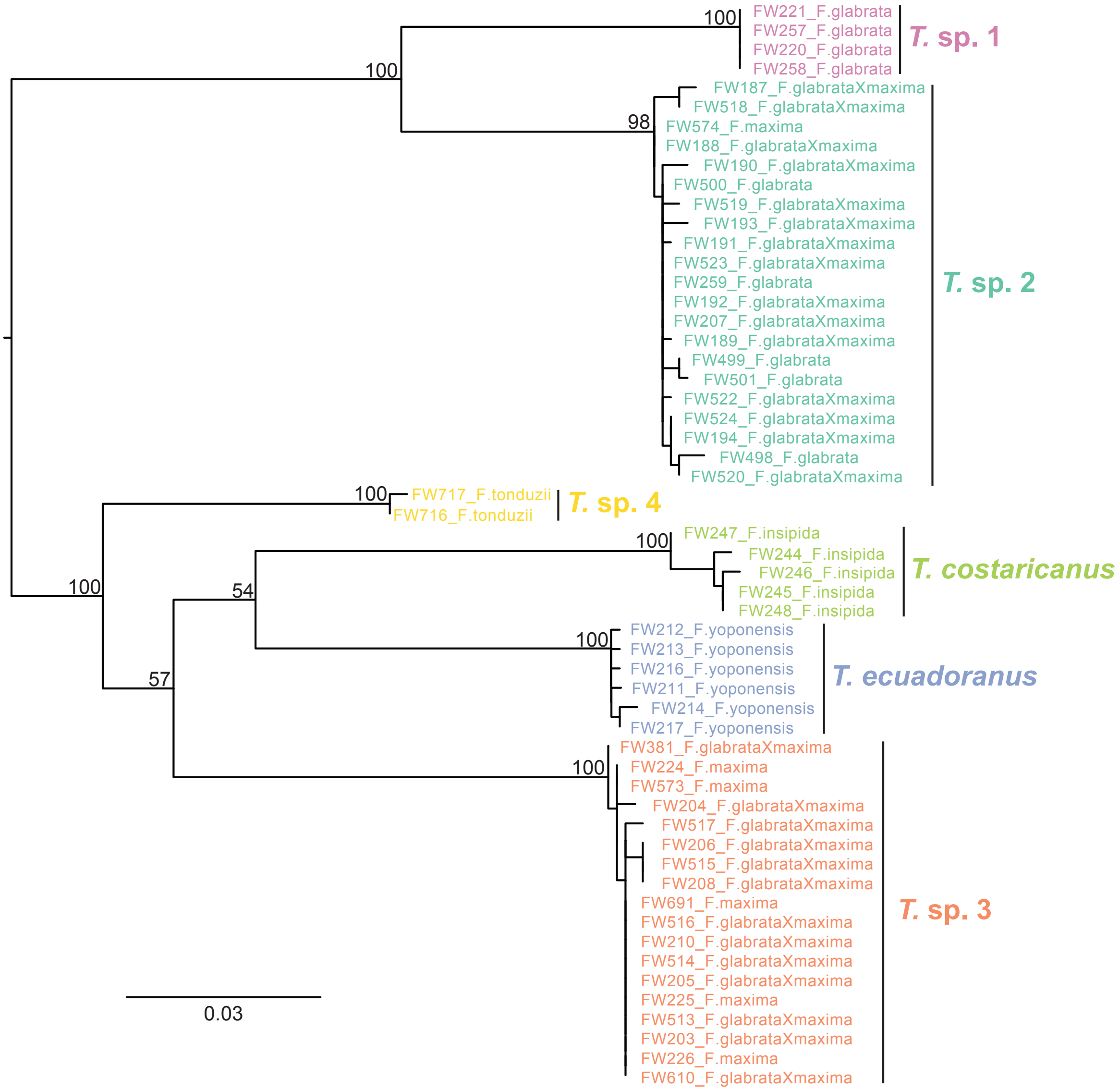

Figure S1: Maximum likelihood gene tree estimate of *Tetrapus* wasp COI data obtained from UCE sequencing. Nodal support values represent bootstrap support. Individuals are labeled with their unique identifier (FW#) and the host species from which they were sampled. The tree was midpoint rooted for visual purposes.

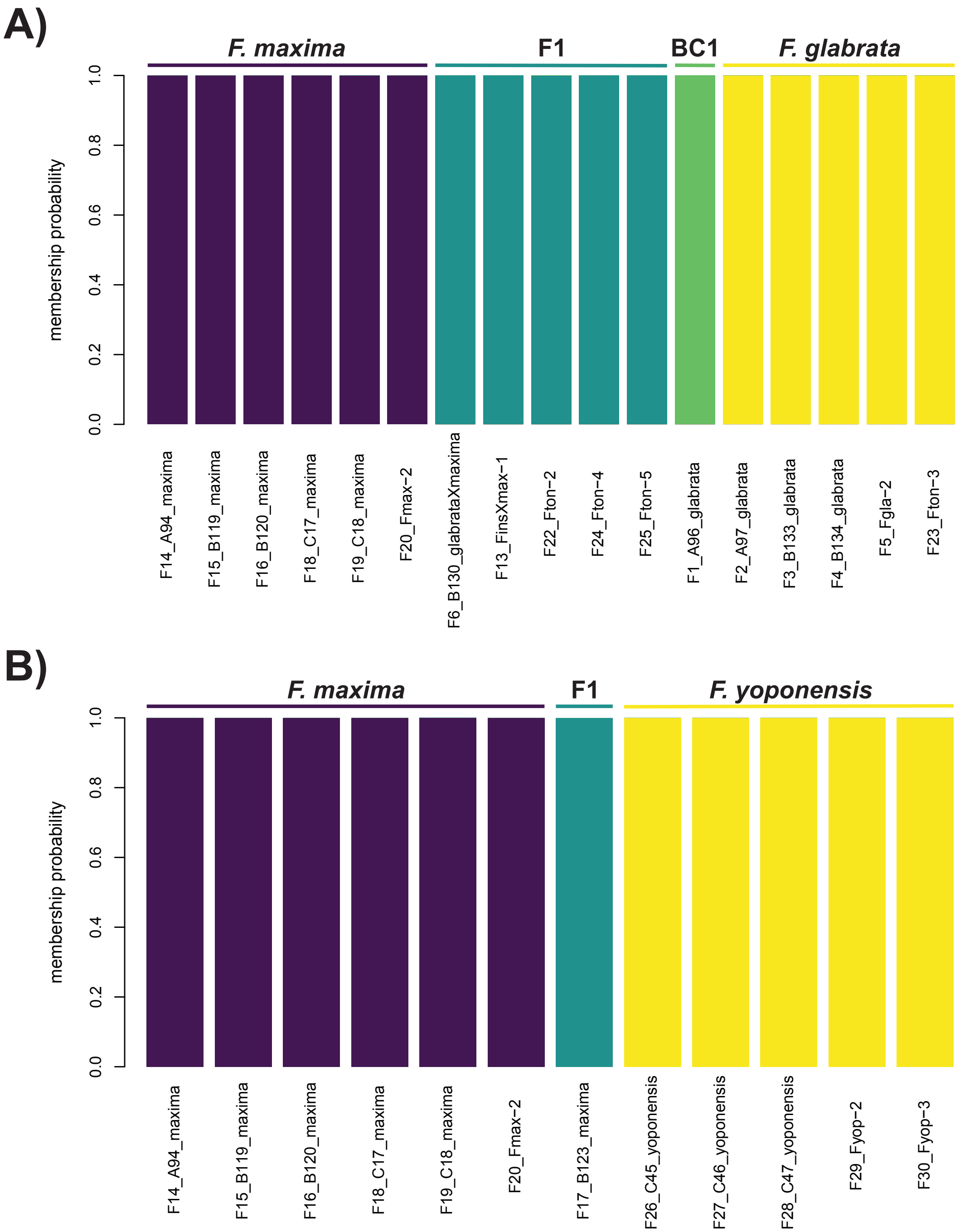

Figure S2: Hybrid analyses with snapclust. Panel A shows hybrids identified between *F. glabrata* and *F. maxima*, including five F1s and one first-generation backcross (BC1) to *F. glabrata*. Panel B shows an F1 hybrid identified between *F. maxima* and *F. yoponensis*. Individuals are labeled with their unique identifier (F#) and internal sampling code.

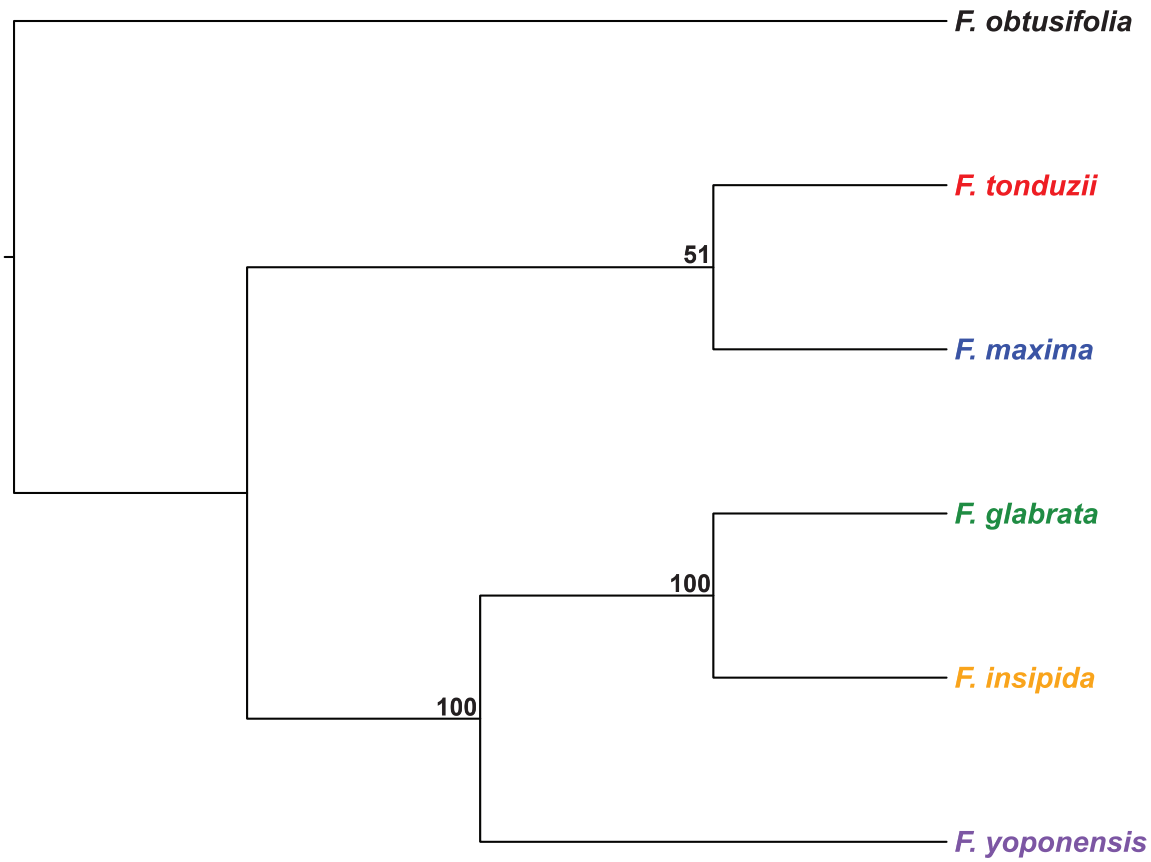

Figure S3: Species tree analysis with SVDQuartets of the *Pharmacosycea* figs. Nodal support values represent bootstrap support.

Individual	Pollinator Wasp	Sampled Host Fig	BioSample
FW187	*Tetrapus* sp. 2	*Ficus glabrata* X *maxima*	SAMN31644000
FW188	*Tetrapus* sp. 2	*Ficus glabrata* X *maxima*	SAMN31644001
FW189	*Tetrapus* sp. 2	*Ficus glabrata* X *maxima*	SAMN31644002
FW190	*Tetrapus* sp. 2	*Ficus glabrata* X *maxima*	SAMN31644003
FW191	*Tetrapus* sp. 2	*Ficus glabrata* X *maxima*	SAMN31644004
FW192	*Tetrapus* sp. 2	*Ficus glabrata* X *maxima*	SAMN31644005
FW193	*Tetrapus* sp. 2	*Ficus glabrata* X *maxima*	SAMN31644006
FW194	*Tetrapus* sp. 2	*Ficus glabrata* X *maxima*	SAMN31644007
FW203	*Tetrapus* sp. 3	*Ficus glabrata* X *maxima*	SAMN31644008
FW204	*Tetrapus* sp. 3	*Ficus glabrata* X *maxima*	SAMN31644009
FW205	*Tetrapus* sp. 3	*Ficus glabrata* X *maxima*	SAMN31644010
FW206	*Tetrapus* sp. 3	*Ficus glabrata* X *maxima*	SAMN31644011
FW207	*Tetrapus* sp. 2	*Ficus glabrata* X *maxima*	SAMN31644012
FW208	*Tetrapus* sp. 3	*Ficus glabrata* X *maxima*	SAMN31644013
FW210	*Tetrapus* sp. 3	*Ficus glabrata* X *maxima*	SAMN31644014
FW211	*Tetrapus ecuadoranus*	*Ficus yoponensis*	SAMN31644015
FW212	*Tetrapus ecuadoranus*	*Ficus yoponensis*	SAMN31644016
FW213	*Tetrapus ecuadoranus*	*Ficus yoponensis*	SAMN31644017
FW214	*Tetrapus ecuadoranus*	*Ficus yoponensis*	SAMN31644018
FW216	*Tetrapus ecuadoranus*	*Ficus yoponensis*	SAMN31644019
FW217	*Tetrapus ecuadoranus*	*Ficus yoponensis*	SAMN31644020
FW220	*Tetrapus* sp. 1	*Ficus glabrata*	SAMN31644021
FW221	*Tetrapus* sp. 1	*Ficus glabrata*	SAMN31644022
FW224	*Tetrapus* sp. 3	*Ficus maxima*	SAMN31644023
FW225	*Tetrapus* sp. 3	*Ficus maxima*	SAMN31644024
FW226	*Tetrapus* sp. 3	*Ficus maxima*	SAMN31644025
FW244	*Tetrapus costaricanus*	*Ficus insipida*	SAMN31644026
FW245	*Tetrapus costaricanus*	*Ficus insipida*	SAMN31644027
FW246	*Tetrapus costaricanus*	*Ficus insipida*	SAMN31644028
FW247	*Tetrapus costaricanus*	*Ficus insipida*	SAMN31644029
FW248	*Tetrapus costaricanus*	*Ficus insipida*	SAMN31644030
FW257	*Tetrapus* sp. 1	*Ficus glabrata*	SAMN31644031
FW258	*Tetrapus* sp. 1	*Ficus glabrata*	SAMN31644032
FW259	*Tetrapus* sp. 2	*Ficus glabrata*	SAMN31644033
FW381	*Tetrapus* sp. 3	*Ficus glabrata* X *maxima*	SAMN31644034
FW498	*Tetrapus* sp. 2	*Ficus glabrata*	SAMN31644035
FW499	*Tetrapus* sp. 2	*Ficus glabrata*	SAMN31644036
FW500	*Tetrapus* sp. 2	*Ficus glabrata*	SAMN31644037
FW501	*Tetrapus* sp. 2	*Ficus glabrata*	SAMN31644038
FW513	*Tetrapus* sp. 3	*Ficus glabrata* X *maxima*	SAMN31644039
FW514	*Tetrapus* sp. 3	*Ficus glabrata* X *maxima*	SAMN31644040

FW515	*Tetrapus* sp. 3	*Ficus glabrata* X *maxima*	SAMN31644041
FW516	*Tetrapus* sp. 3	*Ficus glabrata* X *maxima*	SAMN31644042
FW517	*Tetrapus* sp. 3	*Ficus glabrata* X *maxima*	SAMN31644043
FW518	*Tetrapus* sp. 2	*Ficus glabrata* X *maxima*	SAMN31644044
FW519	*Tetrapus* sp. 2	*Ficus glabrata* X *maxima*	SAMN31644045
FW520	*Tetrapus* sp. 2	*Ficus glabrata* X *maxima*	SAMN31644046
FW521	*Tetrapus* sp. 2	*Ficus glabrata* X *maxima*	SAMN31644047
FW522	*Tetrapus* sp. 2	*Ficus glabrata* X *maxima*	SAMN31644048
FW523	*Tetrapus* sp. 2	*Ficus glabrata* X *maxima*	SAMN31644049
FW524	*Tetrapus* sp. 2	*Ficus glabrata* X *maxima*	SAMN31644050
FW573	*Tetrapus* sp. 3	*Ficus maxima*	SAMN31644051
FW574	*Tetrapus* sp. 2	*Ficus maxima*	SAMN31644052
FW610	*Tetrapus* sp. 3	*Ficus glabrata* X *maxima*	SAMN31644053
FW691	*Tetrapus* sp. 3	*Ficus maxima*	SAMN31644054
FW716	*Tetrapus* sp. 4	*Ficus tonduzii*	SAMN31644055
FW717	*Tetrapus* sp. 4	*Ficus tonduzii*	SAMN31644056

Table S1: Pollinator wasp sampling. Information includes unique individual identifier (FW#), pollinator wasp species, sampled host fig species, and NCBI BioSample accession number.

**Individual**	**Sampled Host Fig**	**BioSample**
F1	*Ficus glabrata* X *maxima*	SAMN31644057
F2	*Ficus glabrata*	SAMN31644058
F3	*Ficus glabrata*	SAMN31644059
F4	*Ficus glabrata*	SAMN31644060
F5	*Ficus glabrata*	SAMN31644061
F6	*Ficus glabrata* X *maxima*	SAMN31644062
F7	*Ficus insipida*	SAMN31644063
F8	*Ficus insipida*	SAMN31644064
F9	*Ficus insipida*	SAMN31644065
F10	*Ficus insipida*	SAMN31644066
F11	*Ficus insipida*	SAMN31644067
F12	*Ficus insipida*	SAMN31644068
F13	*Ficus glabrata* X *maxima*	SAMN31644069
F14	*Ficus maxima*	SAMN31644070
F15	*Ficus maxima*	SAMN31644071
F16	*Ficus maxima*	SAMN31644072
F17	*Ficus maxima* X *yoponensis*	SAMN31644073
F18	*Ficus maxima*	SAMN31644074
F19	*Ficus maxima*	SAMN31644075
F20	*Ficus maxima*	SAMN31644076
F21	*Ficus tonduzii*	SAMN31644077
F22	*Ficus glabrata* X *maxima*	SAMN31644078
F23	*Ficus glabrata*	SAMN31644079
F24	*Ficus glabrata* X *maxima*	SAMN31644080
F25	*Ficus glabrata* X *maxima*	SAMN31644081
F26	*Ficus yoponensis*	SAMN31644082
F27	*Ficus yoponensis*	SAMN31644083
F28	*Ficus yoponensis*	SAMN31644084
F29	*Ficus yoponensis*	SAMN31644085
F30	*Ficus yoponensis*	SAMN31644086

Table S2: Host fig sampling. Information includes unique individual identifier (F#), host fig species, and NCBI BioSample accession number.

Individual	Pollinator Wasp	Raw Reads	Contigs	Loci
FW220	*Tetrapus* sp. 1	4687114	238899	1560
FW221	*Tetrapus* sp. 1	4761416	215821	1549
FW257	*Tetrapus* sp. 1	4185059	215301	1547
FW258	*Tetrapus* sp. 1	3228608	155607	1481
FW187	*Tetrapus* sp. 2	3568557	169184	1504
FW188	*Tetrapus* sp. 2	5395745	252736	1629
FW189	*Tetrapus* sp. 2	2952646	150594	1462
FW190	*Tetrapus* sp. 2	3359756	170233	1547
FW191	*Tetrapus* sp. 2	4498239	219901	1612
FW192	*Tetrapus* sp. 2	2595606	125815	1441
FW193	*Tetrapus* sp. 2	4454112	216000	1572
FW194	*Tetrapus* sp. 2	3419353	169235	1511
FW207	*Tetrapus* sp. 2	2538297	140069	1458
FW259	*Tetrapus* sp. 2	3038800	161362	1451
FW498	*Tetrapus* sp. 2	1945324	97408	1333
FW499	*Tetrapus* sp. 2	1870326	89661	1338
FW500	*Tetrapus* sp. 2	2210143	115297	1386
FW501	*Tetrapus* sp. 2	2368081	128566	1399
FW518	*Tetrapus* sp. 2	2568465	147908	1484
FW519	*Tetrapus* sp. 2	2205107	117843	1463
FW520	*Tetrapus* sp. 2	1510105	72769	1301
FW521	*Tetrapus* sp. 2	1471472	66184	1260
FW522	*Tetrapus* sp. 2	1747608	85640	1298
FW523	*Tetrapus* sp. 2	1818483	92336	1323
FW524	*Tetrapus* sp. 2	1719095	78653	1298
FW574	*Tetrapus* sp. 2	1605183	78770	1321
FW244	*Tetrapus costaricanus*	3135303	145513	1409
FW245	*Tetrapus costaricanus*	4301642	194341	1440
FW246	*Tetrapus costaricanus*	4520791	211143	1471
FW247	*Tetrapus costaricanus*	2918422	132518	1356
FW248	*Tetrapus costaricanus*	5135872	247608	1531
FW203	*Tetrapus* sp. 3	1490824	62516	1295
FW204	*Tetrapus* sp. 3	1680263	78249	1315
FW205	*Tetrapus* sp. 3	1746078	84054	1308
FW206	*Tetrapus* sp. 3	1657271	74874	1270
FW208	*Tetrapus* sp. 3	1410770	58561	1244
FW210	*Tetrapus* sp. 3	2069568	106515	1360
FW224	*Tetrapus* sp. 3	3025803	146263	1428
FW225	*Tetrapus* sp. 3	4063502	205176	1549
FW226	*Tetrapus* sp. 3	3968433	201191	1509
FW381	*Tetrapus* sp. 3	2141143	105894	1334

FW513	*Tetrapus* sp. 3	2335878	122856	1414
FW514	*Tetrapus* sp. 3	864467	5561	650
FW515	*Tetrapus* sp. 3	1434738	59929	1303
FW516	*Tetrapus* sp. 3	1628628	72400	1308
FW517	*Tetrapus* sp. 3	2079692	104920	1373
FW573	*Tetrapus* sp. 3	2384204	124031	1383
FW610	*Tetrapus* sp. 3	8810987	331314	1607
FW691	*Tetrapus* sp. 3	3568872	196911	1467
FW716	*Tetrapus* sp. 4	7259025	314992	1655
FW717	*Tetrapus* sp. 4	3560025	191594	1432
FW211	*Tetrapus ecuadoranus*	3474554	189134	1508
FW212	*Tetrapus ecuadoranus*	6117746	320937	1643
FW213	*Tetrapus ecuadoranus*	6353427	320962	1611
FW214	*Tetrapus ecuadoranus*	3144128	162576	1454
FW216	*Tetrapus ecuadoranus*	2816255	146790	1360
FW217	*Tetrapus ecuadoranus*	5795350	307336	1579

Table S3: Pollinator wasp sequencing. Information includes unique individual identifier (FW#), pollinator wasp species, number of raw sequence reads, number of Trinity contigs, and number of UCE loci.

**Individual**	**Host Fig**	**Raw Reads**	**Clusters**	**Loci**
F1	*Ficus glabrata* X *maxima*	7244559	176519	32601
F2	*Ficus glabrata*	4887963	165416	24792
F3	*Ficus glabrata*	4214450	63549	34655
F4	*Ficus glabrata*	7119332	72796	36369
F5	*Ficus glabrata*	2583903	60137	20939
F6	*Ficus glabrata* X *maxima*	4060762	67775	35566
F7	*Ficus insipida*	1003795	44201	16507
F8	*Ficus insipida*	7909847	76907	36970
F9	*Ficus insipida*	2017468	50610	29427
F10	*Ficus insipida*	6030721	74683	36611
F11	*Ficus insipida*	6928368	67783	36858
F12	*Ficus insipida*	7347462	77847	37166
F13	*Ficus glabrata* X *maxima*	1772506	36250	16900
F14	*Ficus maxima*	2082861	53092	29079
F15	*Ficus maxima*	2054747	63114	27703
F16	*Ficus maxima*	2311959	57340	31056
F17	*Ficus maxima* X *yoponensis*	1547830	47958	23657
F18	*Ficus maxima*	24089307	93134	37423
F19	*Ficus maxima*	15711473	81113	37464
F20	*Ficus maxima*	1917990	40238	17481
F21	*Ficus tonduzii*	20840966	116802	36924
F22	*Ficus glabrata* X *maxima*	15431214	86425	37364
F23	*Ficus glabrata*	19882495	92835	37502
F24	*Ficus glabrata* X *maxima*	17770360	95279	37868
F25	*Ficus glabrata* X *maxima*	1947746	37936	18174
F26	*Ficus yoponensis*	25289469	97790	37606
F27	*Ficus yoponensis*	17247374	84773	37546
F28	*Ficus yoponensis*	10196819	74354	37302
F29	*Ficus yoponensis*	10046758	70781	35725
F30	*Ficus yoponensis*	12440898	78933	36314

Table S4: Host fig sequencing. Information includes unique individual identifier (F#), host fig species, number of raw sequence reads, number of RAD clusters, and number of RAD loci when requiring at least 50% sampling presence.
